# Long‐Term Safety of Extremely Low‐Dose Amiodarone at 50 mg Daily in Patients With Persistent Atrial Fibrillation

**DOI:** 10.1002/joa3.70150

**Published:** 2025-07-17

**Authors:** Kentaro Yoshida, Yuta Okabe, Masako Baba, Ko Funabashi, Mami Narita, Shunsuke Kuchitsu, Akinori Sugano, Hideyuki Hasebe, Tomoko Ishizu, Noriyuki Takeyasu

**Affiliations:** ^1^ Department of Cardiology, Institute of Medicine University of Tsukuba Tsukuba Japan; ^2^ Department of Cardiology Ibaraki Prefectural Central Hospital Kasama Japan

**Keywords:** amiodarone, atrial fibrillation, interstitial pneumonia, KL‐6, side effect

## Abstract

**Background:**

Rhythm control of atrial fibrillation (AF) by pulmonary vein isolation alone is commonly difficult in this aging society, and the role of pharmacological therapy is being revisited. Identifying the lowest dose of amiodarone is important as this drug causes dose‐ and duration‐related lung toxicity. Long‐term safety of the use of extremely low‐dose amiodarone in Japanese patients was retrospectively evaluated.

**Methods:**

Included were 120 patients treated with extremely low‐dose amiodarone (50 mg daily) for persistent AF. KL‐6 level was systematically measured at baseline and every 3 months thereafter. The patients were classified into a different quartiles (Q) based on the KL‐6 level measured at baseline (Q1, Q2, Q3, and Q4). Thyroid function was also evaluated at each follow‐up.

**Results:**

During a mean follow‐up period of 51 months, KL‐6 elevation (> 700 U/mL) occurred in 7 (5.8%) patients with higher baseline KL‐6 (Q1, 0 patients; Q2, 0 patients; Q3, 1 patient; and Q4, 6 patients; *p* = 0.0018). Interstitial pneumonia (IP) was diagnosed in 1 (0.8%) patient in Q3 who recovered without the use of steroids. ROC curve analysis showed a cut‐off value for KL‐6 of 283 U/mL for predicting the subsequent elevation. Approximately 70% of the patients were free from recurrence of AF, although electrical cardioversion was required to restore sinus rhythm in 58 (48%) of them.

**Conclusions:**

Even an extremely low dose of amiodarone may potentially contribute to maintenance of sinus rhythm in highly selected patients with persistent AF. A low baseline KL‐6 level may indicate patients at lower risk for amiodarone‐related IP.

## Graphical abstract

The use of extremely low‐dose amiodarone (50 mg once daily) was safe and effective in Japanese patients with persistent AF. A low baseline KL‐6 level may indicate patients at lower risk for amiodarone‐related interstitial pneumonia.

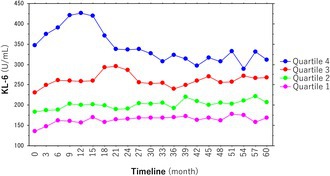

## 1 Introduction

A quarter century has passed since the introduction of pulmonary vein (PV) isolation as a nonpharmacological treatment of atrial fibrillation (AF). Thanks to advancements in technologies, such as three‐dimensional mapping systems, irrigation‐tip catheters, contact force monitoring, balloon ablation, and pulsed‐field ablation, safety, and efficacy for acute outcomes have been improved. However, electrical reconnections between the PVs and left atrium in the chronic phase after ablation are still common, and thus, a redo procedure is required in one‐third of the patients. More importantly, especially in persistent AF, additional ablation beyond the PVs is not standardized, and the efficacy of ablation targeting the substrate of persistent AF is not satisfactory [1, 2].

With this limitation of ablation therapy and the increase in older patients with heart failure due to AF and diastolic dysfunction, that is, heart failure with preserved ejection fraction, pharmacological therapy should be revisited. For a decade, we commonly prescribed an extremely low dose of amiodarone (50 mg daily) in patients both with and without a history of catheter ablation and systematically measured the serum level of Krebs von den Lungen‐6 (KL‐6) every 3 months for early detection of the possible side effect of interstitial pneumonia (IP). In this study, the long‐term efficacy and safety of the use of extremely low‐dose amiodarone in Japanese patients were retrospectively evaluated, especially by focusing on the systematic measurements of KL‐6.

## 2 Methods

### 2.1 Study Subjects

We retrospectively reviewed cases of patients treated with extremely low‐dose amiodarone (50 mg once daily) for persistent AF in our institution from April 2016 to December 2022. No patients with lung disease such as severe chronic obstructive pulmonary disease, idiopathic or collagen disease‐related IP, and lung cancer took amiodarone. We identified 120 patients who had undergone systematic measurements of KL‐6 before the introduction of amiodarone (baseline KL‐6) and every 3 months thereafter at the outpatient clinic [3]. The loading dose for 2 weeks was 100 mg in 34 (28%), 200 mg in 74 (62%), and 400 mg in 12 (10%) patients. Patients taking a higher dose of amiodarone for ventricular tachyarrhythmias were not included in the study according to the study purpose. The patients were classified into a quartile (Q) of their KL‐6 value at baseline (Q1, Q2, Q3, and Q4). The normal value of KL‐6 was < 450 U/mL (BML Inc., Tokyo), and significant elevation of KL‐6 during follow‐up was defined as > 700 U/mL in the present study. Patients with elevation of KL‐6 also underwent computed tomography (CT) scan of the lung to rule IP out or in. Additionally, thyroid stimulating hormone, FT4, and FT3 were measured to detect thyroid function at the same timing of KL‐6 measurements. The study protocol was approved by the local institutional review board of Ibaraki Prefectural Central Hospital (approval no. 1452) and complied with the Declaration of Helsinki. The opt‐out method announcing the handling of personal data and the right to withdraw consent was shown on the website of the institution.

### 2.2 Catheter Ablation and Subsequent Follow‐Up

The extremely low dose of amiodarone was commonly prescribed for the recurrence of AF after catheter ablation in 80 (67%) patients. The details of catheter ablation were described previously [4]. Briefly, the ipsilateral PV antrum was circumferentially ablated under fluoroscopic, electrogram, and three‐dimensional electro‐anatomic mapping system (CARTO, Biosense Webster, Diamond Bar, CA) guidance. Radiofrequency energy was delivered at a power of 20–35 W and at a target ablation index of 450 at the anterior aspect and of 350–400 at the posterior aspect (ThermoCool *SmartTouch*, Biosense Webster). Additional approaches to PV isolation, such as posterior wall isolation and linear ablation, were performed according to the operator's discretion. During follow‐up, recurrence of AF was defined as lasting > 30 s and detected by 12‐lead ECG at the outpatient clinic and/or 24‐h Holter electrocardiogram.

### 2.3 Statistical Analysis

Continuous variables are expressed as mean ± SD or median [interquartile range], and categorical variables are reported as number and percentage. Statistical differences were tested using an unpaired Student *t*‐test, Mann–Whitney *U* test, or chi‐squared analysis, as appropriate. One‐way analysis of variance (ANOVA) was used to compare the continuous results from the four groups (Q1, Q2, Q3, and Q4). A receiver operating characteristic (ROC) curve was created to determine the possible cutoff value of baseline KL6 for predicting its subsequent elevation. Statistical significance was considered when the *p* value was < 0.05. All statistical analyses were carried out using JMP version 16 (SAS Institute Inc., Cary, NC).

## 3 Results

### 3.1 Patient Characteristics

The mean follow‐up period was 51 months. The baseline patient characteristics of the patients in each Q are summarized in Table 1 and were not significantly different between the four Qs. The baseline KL‐6 was 136 ± 20 in Q1, 183 ± 12 in Q2, 230 ± 20 in Q3, and 348 ± 66 U/mL in Q4.

**TABLE 1:** Baseline clinical characteristics.

Characteristics	All patients (*n* = 120)	Q1 (*n* = 30)	Q2 (*n* = 30)	Q3 (*n* = 30)	Q4 (*n* = 30)	*p*
Baseline KL‐6 (U/mL)	224 ± 87	136 ± 20	183 ± 12	230 ± 20	348 ± 66	—
Female, *n* (%)	48 (40)	16 (53)	10 (33)	14 (47)	8 (27)	0.135
Age (years)	74 ± 12	75 ± 11	73 ± 11	73 ± 13	74 ± 11	0.954
Body mass index (kg/m^2^)	25 ± 5	24.4 ± 6.5	25.1 ± 3.6	25.4 ± 4.3	24.4 ± 4.2	0.813
Body surface area (m^2^)	1.63 ± 0.24	1.58 ± 0.25	1.68 ± 0.23	1.65 ± 0.23	1.64 ± 0.18	0.413
AF duration (months)	4.0 [3.0]	6.0 [5.0]	5.0 [5.0]	3.0 [3.0]	3.0 [5.0]	0.216
History of catheter ablation, *n* (%)	80 (67)	16 (53)	23 (77)	22 (73)	19 (63)	0.212
Comorbidities						
Sick sinus syndrome, *n* (%)	33 (28)	10 (33)	7 (23)	8 (27)	8 (27)	0.875
Hypertension, *n* (%)	74 (62)	16 (53)	20 (67)	22 (73)	16 (53)	0.283
Diabetes, *n* (%)	31 (26)	5 (17)	6 (20)	10 (33)	10 (33)	0.306
Hypertrophic cardiomyopathy, *n* (%)	20 (17)	7 (23)	1 (3)	5 (17)	7 (23)	0.123
Ischemic heart disease, *n* (%)	10 (8)	4 (13)	2 (7)	2 (7)	2 (7)	0.727
Valvular heart disease, *n* (%)	28 (23)	7 (23)	6 (20)	7 (23)	8 (27)	0.945
Echocardiography						
Ejection fraction (%)	61 ± 11	58 ± 15	60 ± 9	63 ± 7	63 ± 9	0.262
LAVi (mL/m^2^)	44 ± 16	49 ± 14	42 ± 17	45 ± 18	41 ± 14	0.195
eGFR (mL/min/1.73 m^2^)	56 ± 16	56 ± 16	58 ± 19	55 ± 16	56 ± 16	0.853
BNP (pg/mL)	68 [103]	128 [184]	54 [72]	66 [88]	60 [81]	0.210

*Note:* AF duration and BNP: median [interquartile range].

Abbreviations: AF, atrial fibrillation; BNP, brain natriuretic peptide; eGFR, estimated glomerular filtration rate; KL‐6, Krebs von den Lungen‐6; LAVi, left atrial volume index; Q, quartile.

### 3.2 Rhythm Outcomes

In 17 (43%) of the patients not undergoing catheter ablation, low‐dose amiodarone was not capable of restoring sinus rhythm from persistent AF, and thus, sinus rhythm was restored by cardioversion. In 41 (51%) of the patients undergoing catheter ablation, cardioversion was performed a median of 5 months postablation, including cardioversion within the blanking period of 2 months in 18 (44%) patients and after the blanking period in the remaining 23 (56%) patients. Amiodarone was initiated 2–4 weeks before planned cardioversion and immediately after unplanned cardioversion. In the remaining 39 (49%) patients, amiodarone alone or atrial antitachycardia pacing (Reactive ATP, Medtronic) for patients with cardiac implantable electronic devices converted AF to sinus rhythm.

The mean number of ablation procedures was 1.7 among the patients with a history of catheter ablation, indicating that the extremely low‐dose amiodarone was most commonly prescribed for rhythm control to patients with AF resistant to multiple catheter ablations. During the follow‐up period, amiodarone was discontinued due to KL‐6 elevation in 7 (5.8%) patients. Among those patients who could continue to take amiodarone 50 mg (*n* = 113), AF recurred in 35 (31%) patients: 30 (86%) patients with an ablation history and 5 (14%) patients with no ablation history (Table 2). In patients with recurrence of AF, an additional ablation procedure (*n* = 4, 11%) and an increase in the amiodarone dosage to 100–200 mg for rhythm control (*n* = 9, 26%) or rate control (*n* = 22, 63%) were applied as subsequent treatment.

**TABLE 2:** Baseline characteristics of the patients with and without a history of ablation.

Characteristics	Ablation (*n* = 80)	No ablation (*n* = 40)	*p*
Female, *n* (%)	32 (40)	16 (40)	1.000
Age (years)	73 ± 10	77 ± 14	0.075
Body mass index (kg/m^2^)	24.9 ± 4.1	24.1 ± 6.0	0.364
AF duration (months)	5.0 [5.5]	4.0 [3.0]	0.322
Comorbidities			
Sick sinus syndrome, *n* (%)	25 (31)	8 (20)	0.193
Hypertension, *n* (%)	53 (66)	21 (53)	0.144
Diabetes, *n* (%)	21 (26)	10 (25)	0.883
Hypertrophic cardiomyopathy, *n* (%)	13 (16)	7 (18)	0.863
Ischemic heart disease, *n* (%)	5 (6)	5 (13)	0.243
Valvular heart disease, *n* (%)	20 (25)	8 (20)	0.542
Echocardiography			
Ejection fraction (%)	63 ± 7	58 ± 16	0.021
LAVi (mL/m^2^)	44 ± 16	45 ± 16	0.903
eGFR (mL/min/1.73 m^2^)	58 ± 16	52 ± 15	0.045
BNP (pg/mL)	56 [86]	107 [176]	0.007

*Note:* AF duration and BNP: median [interquartile range].

Abbreviations: AF, atrial fibrillation; BNP, brain natriuretic peptide; eGFR, estimated glomerular filtration rate; KL‐6, Krebs von den Lungen‐6; LAVi, left atrial volume index.

### 3.3 Elevation of KL‐6 and Interstitial Pneumonia

**FIGURE 1:**
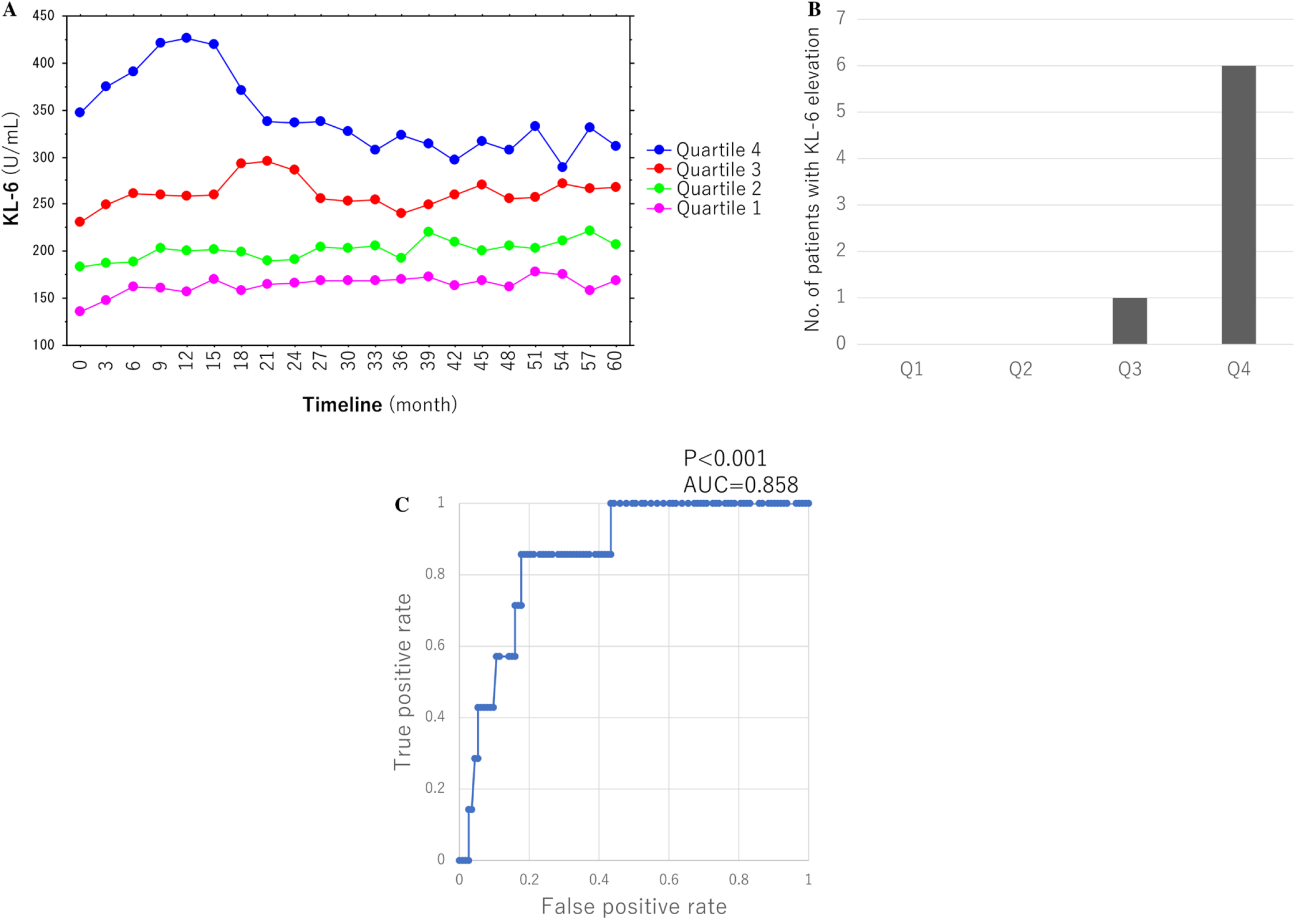
(A) Mean KL‐6 values during follow‐up in each quartile. (B) Number of patients with KL‐6 elevation in each quartile. (C) Receiver operating characteristic curve to determine the cutoff value of baseline KL‐6 for predicting subsequent KL‐6 elevation.

**FIGURE 2:**
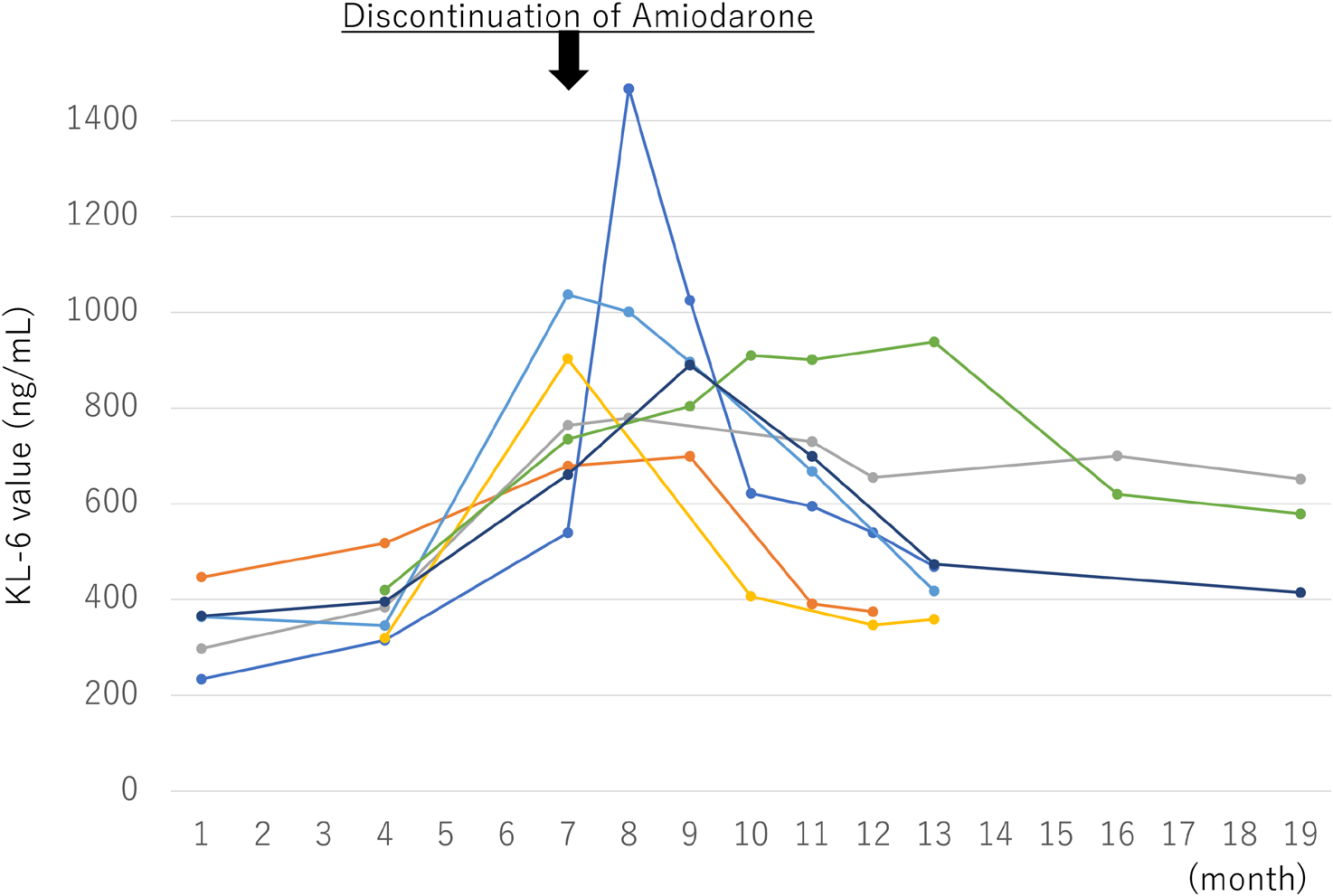
Time course of KL‐6 values in each patient with KL‐6 elevation (*n* = 7).

Significant KL‐6 elevation occurred in 7 (5.8%) patients with higher baseline KL‐6 levels (Q1, 0 patients; Q2, 0 patients; Q3, 1 patient; and Q4, 6 patients; *p* = 0.0018) (Figure 1A,B). IP was diagnosed in 1 (0.8%) patient in Q3 by lung CT scan. The mean duration between the introduction of amiodarone and KL‐6 elevation was 8.6 ± 5.3 months. There were no significant differences in the other baseline characteristics between the patients with and without KL‐6 elevation (Table 3). After discontinuation of amiodarone, the KL‐6 value decreased to the normal limit within 6 months in five patients but remained at a high value > 500 U/mL in two patients (Figure 2). All patients recovered without the use of steroids and without aftereffects, including dyspnea. Two (29%) patients underwent additional catheter ablation after discontinuation of amiodarone and are presently free from recurrence of AF.

**TABLE 3:** Baseline clinical characteristics of the patients with and without KL‐6 elevation.

Characteristics	Elevation (*n* = 7)	No elevation (*n* = 113)	*p*
Baseline KL‐6 (U/mL)	336 ± 82	217 ± 83	< 0.001
Female, *n* (%)	3 (43)	45 (40)	0.874
Age (years)	70 ± 9	74 ± 12	0.402
Body mass index (kg/m^2^)	24.4 ± 5.3	24.7 ± 4.8	0.875
Body surface area (m^2^)	1.63 ± 0.17	1.63 ± 0.24	0.999
AF duration (months)	3.0 [3.5]	4.0 [3.25]	0.376
History of catheter ablation, *n* (%)	6 (86)	74 (65)	0.271
Comorbidities			
Sick sinus syndrome, *n* (%)	3 (43)	30 (27)	0.348
Hypertension, *n* (%)	3 (43)	71 (63)	0.292
Diabetes, *n* (%)	3 (43)	28 (25)	0.289
Hypertrophic cardiomyopathy, *n* (%)	1 (14)	19 (17)	0.862
Ischemic heart disease, *n* (%)	0 (0)	10 (9)	0.411
Valvular heart disease, *n* (%)	4 (57)	24 (21)	0.051
Echocardiography			
Ejection fraction (%)	64 ± 10	61 ± 11	0.470
LAVi (mL/m^2^)	37 ± 8	45 ± 16	0.193
eGFR (mL/min/1.73 m^2^)	56 ± 18	56 ± 16	0.980
BNP (pg/mL)	40 [110]	71 [100]	0.362

*Note:* AF duration and BNP: median [interquartile range].

Abbreviations: AF, atrial fibrillation; BNP, brain natriuretic peptide; eGFR, estimated glomerular filtration rate; KL‐6, Krebs von den Lungen‐6; LAVi, left atrial volume index.

Moreover, the ROC curve created to determine the possible cutoff value of baseline KL6 for predicting its subsequent elevation showed a cutoff value of 283 U/mL and an area under the curve of 0.858 (Figure 1C).

### 3.4 Thyroid Dysfunction

Hypothyroidism was diagnosed in 8 (6.7%) patients 17 ± 11 months after the initiation of amiodarone, and thyroid hormone replacement was begun without discontinuing amiodarone in all patients. Hyperthyroidism was diagnosed in 5 (4.2%) patients 26 ± 20 months after the initiation of amiodarone, following which amiodarone was discontinued and thyroid function normalized in all five patients.

## 4 Discussion

Identifying the lowest dose of amiodarone with antiarrhythmic and hemodynamic activity is important as this drug causes serious dose‐ and duration‐related toxicity. To the best of our knowledge, this is the first study to evaluate the safety and efficacy of extremely low dose, 50 mg daily administration of amiodarone in patients with persistent AF over a long‐term follow‐up period. The potentially important and novel findings of our study are as follows:

1. Although highly selected patients were included, extremely low‐dose amiodarone was modestly effective (freedom from AF recurrence in 60% patients) for ablation‐resistant persistent AF during long‐term follow‐up.
2. Even if extremely low‐dose amiodarone was used, a significant number of patients (6%) suffered from KL‐6 elevation and were obliged to cease taking amiodarone and change their therapy to rate control.
3. KL‐6 elevation during the follow‐up was likely to occur in patients with a higher baseline KL‐6 value, which could be a marker to stratify the risk for KL‐6 elevation and IP.
4. Although a significant number of patients with KL‐6 elevation were observed, no patients suffered from IP that progressed to respiratory dysfunction or required steroid therapy. We hypothesized that this may be an advantage of extremely low‐dose amiodarone, in which immediate cessation of amiodarone can contribute to rapid detoxification before IP develops.

### 4.1 Effectiveness of Low‐Dose Amiodarone in Preventing AF Recurrence

The definition of “low dose” and “very low dose” varies between studies [5]. Because amiodarone was initially prescribed at a higher maintenance dose of > 400 mg daily, “low dose” is commonly defined as < 200 mg daily, which is actually 100 or 200 mg, in the literature. To the best of our knowledge, only one small study was conducted in the 1990s that systematically investigated the short‐term outcomes of amiodarone 50 mg daily (*n* = 15) [6]. These patients with low ejection fraction and ventricular arrhythmias were randomized into placebo, amiodarone 50 mg, and amiodarone 100 mg groups. The 100 mg amiodarone dose was significantly superior to the placebo group at all levels, and even the 50 mg amiodarone group had a statistically significant reduction in the frequency of ventricular premature complexes and couplets at 12 weeks, and the left ventricular ejection fraction and stroke volume index significantly increased when compared with baseline values, proving the pharmaco‐kinetical effectiveness of the 50 mg dose of amiodarone.

The primary purpose of the present study was to evaluate not the effectiveness of but the safety of extremely low‐dose amiodarone therapy because this therapeutic approach was mainly applied to highly selected patients who had recurrence of AF/atrial tachycardia despite multiple ablation procedures, whereas most previous studies investigating the effect of low‐dose amiodarone (100 or 200 mg) included patients undergoing DC cardioversion. However, the present outcomes indicated that low‐dose amiodarone therapy could be an important alternative choice in the current era when the efficacy of ablation of persistent AF is not satisfactory, especially if the incidence of side effects would be lower. Although amiodarone unexpectedly appeared to be more effective in the patients without a history of catheter ablation than in those with it, this may suggest that AF resistant to catheter ablation might also likely be resistant to drug therapy including amiodarone and that the nature and progression of AF influences the outcome of amiodarone therapy as estimated. A previous study reported that “low dose” amiodarone therapy resulted in freedom from recurrence of AF in 60% of the patients with AF and atrial flutter during a mean follow‐up period of 36 months [7]. However, the patients' background characteristics were significantly different from those of our patients. In that study, “low dose” meant a mean dose of approximately 140 mg/day, including a dose of 200 mg/day in some patients, and most of the patients had heart failure with reduced ejection fraction (35% ± 16%).

### 4.2 KL‐6 Elevation After Amiodarone Intake

The prevalence of KL‐6 elevation was not low even with extremely low‐dose amiodarone therapy in the present study, and all patients with KL‐6 elevation needed to discontinue amiodarone. Although a study with a larger number of patients is needed for verification, a promising finding was that KL‐6 elevation was observed mostly in the patients with a high baseline KL‐6 level, that is, Q4. We thought this to be a clinically helpful finding in selecting patients with low probability of IP associated with amiodarone. Previous studies showed that the sensitivity of KL‐6 was not high (~60%) [8, 9], and an appropriate cutoff value of KL‐6 is still unclear [10]. In contrast, a previous study showed the specificity of KL‐6 was higher (91%) than that of the % diffusing capacity of carbon monoxide (DL_CO_) (approximately 70%), and, of interest, the negative predictive value was 92%. Therefore, KL‐6 is considered useful for assessing an indeterminant background of interstitial lung disease *before* amiodarone therapy begins [10]. Based on the present study and such previous assessments, our finding could support that a low baseline KL‐6 level, indicating little background risk associated with IP, might identify patients at low risk for KL‐6 elevation and possible IP.

### 4.3 IP After Amiodarone Intake

Only one (0.8%) patient developed IP diagnosed by lung CT scan during long‐term follow‐up. This rate was lower than that in the previous studies (2%–8%) [10]. Several reasons are considered. First, only patients taking amiodarone for AF were included in this study in line with the study purpose. The study subjects would likely be healthier (NYHA I or II) than patients with severe left ventricular dysfunction and ventricular arrhythmias. Second, any patients with a history of severe chronic obstructive pulmonary disease and IP were not candidates for use of amiodarone in our institution. Third, and most importantly, the lowest dose of 50 mg daily was systematically prescribed. The lowest dose needed to suppress arrhythmias should be taken to minimize the risk of side effects, and amiodarone 50 mg daily may be a possible and reasonable therapy that well balances benefits and risks, especially in patients with low body surface area such as Japanese patients with atrial tachyarrhythmias.

Two possible mechanisms of IP, a direct toxic effect and an immune‐mediated hypersensitivity‐based mechanism, have been suggested [11]. In the present study, of note, KL‐6 elevation occurred within 1 year postamiodarone prescription in all patients except one who developed IP diagnosed by lung CT scan at 15 months of follow‐up. The early appearance of KL‐6 elevation is one of the findings more suggestive of an immune‐mediated hypersensitivity‐based mechanism, and it may be associated with the acritical clinical course after immediate discontinuation of amiodarone in the present patient series. This may also support the immediate cessation of low‐dose amiodarone potentially having an advantage in contributing to rapid detoxification before IP develops when compared to higher‐dose amiodarone therapy.

### 4.4 Prior Studies

A systemic review and meta‐analysis recently focused on side effects in low‐dose amiodarone (< 200 mg) therapy [5]. Ten studies (totaling 901 patients) were included, and the dose‐ and duration‐dependent nature of the side effects was confirmed. “Very‐low‐dose” was defined as < 100 mg, and the lowest dose of 50 mg daily was actually used in only an extremely limited number of patients, including pediatric patients. There was no study focusing on the long‐term effect and safety of amiodarone 50 mg for persistent AF with systematic measurements of KL‐6.

Recently, a retrospective, large, nationwide study assessing the association between consistent low‐dose amiodarone therapy and the occurrence of IP, lung cancer, and all‐cause mortality in patients with AF was published [12]. The “low‐dose” was basically defined as 200 mg daily. During a median follow‐up of 4.2 years, IP was diagnosed in 2.0% of the patients. However, amiodarone exposure was not significantly related to an increased risk of interstitial lung disease (hazard ratio 1.45, *p* = 0.09). Interestingly, all‐cause death occurred in 2185 (18.1%) patients, and amiodarone exposure was related to a lower risk of all‐cause death compared to the non‐exposure group. This study had several limitations associated with the use of a medical records database based on the International Classification of Diseases (ICD). It did not assess a history of catheter ablation and subclinical adverse effects and defined “low‐dose” as a relatively higher dose of 200 mg. However, this study may further encourage an increase in amiodarone use for rhythm control in AF.

In previous studies, age was a highly predictive risk factor of IP, although not in the present study [10]. The reason for this discrepancy is probably related to patient background. The mean patient age of 75 years old in this study was much higher than that of previous studies mainly including patients with ventricular arrhythmias. Because AF is prevalent in older adults, the findings of our study may basically be intended for patients at higher risk for IP in this regard.

## 5 Limitations

As limitations of this study, the sample size was small, and rhythm outcomes were assessed only by the outpatient clinic and 24‐h Holter recordings. This may have caused under‐detection of the arrhythmia events and overestimation of the efficacy of low‐dose amiodarone therapy. Also, selection bias may be significant, especially in evaluating the efficacy of amiodarone therapy because this was a retrospective study primarily designed to evaluate the safety of rather than the efficacy of amiodarone therapy. Patients with severe heart failure, structural heart disease, and obesity may have been less likely to be candidates for low‐dose amiodarone therapy, and we may have been more eager to maintain sinus rhythm with no or little medication in patients undergoing catheter ablation than in those not undergoing it.

A pulmonary function test based on DL_CO_ was not systematically performed due to the COVID‐19 pandemic during the study period. Susceptibility to IP may be different among populations and races, and body weight should be taken into consideration when determining the dose of amiodarone because the present results derived from Japanese patients with small body size.

## 6 Conclusion

Even an extremely low dose of amiodarone may potentially contribute to the maintenance of sinus rhythm in highly selected patients with persistent AF, although electrical cardioversion was required to restore sinus rhythm in half of the patients. A low baseline KL‐6 level may indicate patients at lower risk for amiodarone‐related IP. Although KL‐6 elevation was observed in a significant number of patients, none suffered from IP progressing to respiratory dysfunction and requiring steroid therapy. In extremely low‐dose amiodarone therapy, immediate cessation of amiodarone based on the serial measurements of KL‐6 may contribute to early and rapid detoxification of amiodarone before IP develops.

## Disclosure

The authors have nothing to report.

## Ethics Statement

The local institutional review board of Ibaraki Prefectural Central Hospital (approval no. 1452).

## Consent

The opt‐out method announcing the handling of personal data and the right to withdraw consent was shown on the website of the institution.

## Conflicts of Interest

The authors declare no conflicts of interest.

## Data Availability

The deidentified participant data will not be shared.
